# The effect of audiovisual feedback of monitor/defibrillators on percentage of appropriate compression depth and rate during cardiopulmonary resuscitation

**DOI:** 10.1186/s12871-023-02304-9

**Published:** 2023-10-05

**Authors:** Hannah Lee, Jay Kim, Somin Joo, Sang-Hoon Na, Sangmin Lee, Sang-Bae Ko, Jinwoo Lee, Seung-Young Oh, Eun Jin Ha, Ho Geol Ryu

**Affiliations:** 1 Department of Anesthesiology and Pain Medicine, Seoul National University Hospital, Seoul National University College of Medicine, Daehak-Ro 101, Jongno-Gu, Seoul, 03080 Republic of Korea; 2 Graduate School of Medical Science and Engineering, Korea Advanced Institute of Science and Technology, Daejeon, Republic of Korea; 3 Division of Cardiology, Department of Internal Medicine, Seoul National University Hospital, Seoul, Korea; 4 Division of Pulmonary and Critical Care Medicine, Department of Internal Medicine, Seoul National University Hospital, Seoul, Korea; 5 Department of Neurology, Seoul National University Hospital, Seoul, Korea; 6 Department of Surgery, Seoul National University Hospital, Seoul, Korea; 7 Department of Neurosurgery, Seoul National University Hospital, Seoul, Korea

**Keywords:** Feedback device, Cardiopulmonary resuscitation, Compression depth

## Abstract

**Background:**

High quality cardiopulmonary resuscitation (CPR) is one of the key elements of the survival chain in cardiac arrest. Audiovisual feedback of chest compressions have been suggested to be beneficial by increasing the quality of CPR in the simulated cardiac arrests.

**Methods:**

A prospective before and after study was performed to investigate the effect of a real-time audiovisual feedback system on CPR quality during in-hospital cardiac arrest in intensive care units from November 2018 to February 2022. In the feedback period, CPR was performed with the aid of the real-time audiovisual feedback system. The primary outcome was the percentage of compressions with both adequate depth (5.0–6.0 cm) and rate (100–120/minute).

**Results:**

A total of 27,295 compressions in 30 cardiac arrests in the no-feedback period and 27,965 compressions in 30 arrests in the feedback period were analyzed. The percentage of compressions with both adequate depth and rate was 11.8% in the feedback period and 16.8% in the no-feedback period (*P* < 0.01). The percentage of compressions with adequate rate in the feedback period was lower than that in the no-feedback period (67.3% vs. 75.5%, *P* < 0.01). The percentage of beyond-target depth with the feedback was significantly higher than that without feedback (64.2% vs. 51.4%, *P* < 0.01).

**Conclusion:**

Real-time audiovisual feedback system did not increase CPR quality and was associated with a higher percentage of compression depth deeper than the recommended 5.0–6.0 cm. It is essential to explore more effective ways of implementing feedback in real clinical settings to improve of the quality of CPR.

**Trial registration:**

NCT03902873 (study start: Nov. 2018, initial release April 2019, retrospectively registered).

**Supplementary Information:**

The online version contains supplementary material available at 10.1186/s12871-023-02304-9.

## Background

The rapid initiation and delivery of high quality chest compressions and defibrillation is key to survival in cardiac arrest [1]. However, healthcare professionals experience physical fatigue while delivering resuscitation, leading to suboptimal quality of cardiopulmonary resuscitation (CPR) [2].

Previous studies showed that CPR is often delivered with suboptimal quality even when healthcare professionals are providing the resuscitation [3, 4]. Numerous studies have focused on finding ways to improve and optimize CPR quality. Real-time audiovisual feedback has been suggested to be effective when applied to simulated cardiac arrests [5, 6]. The most recent American Heart Association guideline for CPR and Emergency Cardiovascular Care (ECC) states that it may be reasonable to use real-time audiovisual feedback device in actual cardiac arrest situations for quality improvement [7].

A recent meta-analysis showed that effect of real-time CPR feedback devices remains controversial with conflicting results regarding return of spontaneous circulation (ROSC) and short-term survival [8]. The effectiveness of CPR feedback devices were variable depending on the type of the devices [8]. Two randomized controlled trials showed that a portable non-automated external defibrillator (non-AED) feedback device (Cardio First Angel™, CFA; INOTECH, Neuberg, Germany) improved sustained ROSC and survival in patients who experienced in-hospital cardiac arrests (IHCA) [9, 10]. However, specific CPR quality measures such as depth, rate, and recoil were not presented and visual feedback was not available. Moreover, there are only a few studies evaluating the effectiveness of a real-time audiovisual feedback system on CPR quality during IHCA [11, 12].

Therefore, we aimed to investigate the effect of real-time audiovisual feedback on various measures of CPR quality, including compression depth, compression rate, and release velocity.

## Methods

This study was a single center prospective before and after study. The institutional review board (IRB) of Seoul National University Hospital approved study protocol (IRB No 1803–065-929) and waived written informed consent due to the emergency circumstances of CPR. All procedures were conducted in accordance with the ethical standards of the committee responsible for human experimentation and the latest version of the Helsinki Declaration.

### Patients and baseline data

Adult patients (> 18 years) who received CPR due to cardiac arrest between November 2018 and February 2022 in the medical or surgical intensive care unit (ICU)s were enrolled. Patients with written form of Physician’s Order of Life Sustaining Treatment, younger or equal to 18-year-old, or who were undergoing CPR outside medical or surgical ICUs were excluded. Demographic data, comorbidities, location of cardiac arrest, initial rhythm (ventricular fibrillation (VF)/pulseless ventricular tachycardia (VT), pulseless electrical activity (PEA), asystole), and CPR duration were recorded.

### CPR team

The CPR team consisted of internal medicine residents on rotation in the medical ICU, a 3rd year anesthesiology resident in the surgical ICU, interns, and experienced ICU nurses. For CPRs performed on patients in the surgical ICU, the on-duty surgical resident in the surgical ICU would join the team. In a typical CPR case, around 10 or more interns and residents participate. Typically, the role of CPR leader was assumed by the resident or the ICU fellow. During periods without feedback, the compressor performed compressions without the aid of audiovisual feedback. During the feedback periods, the compressor carried out compressions while both watching and listening to the audiovisual feedback. Chest compressions were mostly performed by interns, alternating every 2 min to prevent fatigue in accordance with the relevant guidelines. CPR training includes basic life support training and practice, including chest compression one week before starting the internship. Residents are required to participate in Korean Advanced Life Support simulation education every two years, usually in their first and third year.

### Study design

CPR pads (OneStep™, Chelmsford, Massachusetts, USA) were attached to the patient at the beginning of CPR and were connected to the defibrillator (ZOLL R Series® Chelmsford, Massachusetts, USA). The accelerometer was placed on the patient’s lower half of sternum and captured the compression depth, rate, release velocity of every chest compression and the captured information is sent to the defibrillator and the processed data in the defibrillator is provided in real time to the rescuer.

There are two types of audio feedback: a voice prompt that either indicates 'good compression' or instructs to 'push harder', and a metronome sound that beeps when the compression rate falls below 100/min. Visual feedback was displayed on the CPR dashboard of the defibrillator. Depth and rate of compressions, release velocity, and a diamond-shaped perfusion performance indicator was displayed in real time. When the rate or depth of compressions were not within the guideline recommendations, it was displayed on the CPR dashboard with a yellow backlight.

Chest compressions, ventilation, defibrillation, and drug administration were all conducted in accordance with the 2015 CPR guidelines [13]. During the first month of the study before patient enrollment, residents and nurses were trained/educated in use of the CPR pads, defibrillator, and interpretation of feedbacks. Additionally, training sessions on audiovisual feedback were routinely provided to interns and residents leading the CPR team during the initial week of each month. Real-time audiovisual feedback was provided during the feedback period (November 2019 to June 2021, 20 months), but not during the no-feedback period (November 2018 to October 2019 and July 2021 to February 2022, 20 months). Collecting a dataset of 10 cases took a span of one year, from November 2018 to October 2019. We anticipated that if there was a substantial time gap between the periods designated for feedback and no-feedback comparisons, the results could potentially reflect differences arising not only from feedback variations but also from other evolving factors. To mitigate this, we initiated the feedback period one year later, following which we reinstated the no-feedback period to maintain a balanced approach in our study.

### Data analysis

Individual compressions including depth (cm), rate (/min), and release velocity (mm/sec) for the first 10 min of CPR were analyzed. A previous study found a 6.2% decrease in chest wall stiffness over 1,000 compressions [14]. Additionally, adherence to CPR guidelines can be affected by the chest wall's pliability [15]. Given these observations, we chose to focus our analysis on the initial 10 min. In patients who experienced multiple cardiac arrests, only the first event was included for analysis. The target compression rate was 100–120/min and the target compression depth was 5.0–6.0 cm. Chest compression fraction (CCF), defined as the cumulative time spent delivering chest compressions divided by the total resuscitation time [16], was also recorded. To analyze the trends in depth and rate of chest compressions and chest compression release velocity (CCRV) within a single 2 min CPR cycle, we divided each 2-min cycle into four intervals of equal length, each spanning 30 s, facilitating a comparative analysis between these segments.

### Outcomes

The primary outcome was the percentage of compressions with both adequate rate (100 ~ 120/min) and depth (5.0–6.0 cm). Secondary outcomes were the percentage of chest compressions with adequate depth, the percentage of chest compressions with adequate rate, CCRV, rate of return of spontaneous circulation (ROSC), and rate of survival to discharge. Compression depth was grouped shallow (< 5.0 cm), adequate (5.0–6.0 cm), and deep (> 6.0 cm). Compression rate was also grouped into slow (< 100 /min), adequate (100–120 /min), and fast (> 120 /min). In addition, CCRV was grouped into more than or equal to 400 mm/sec, 300–399 mm/sec, and less than 300 mm/sec.

### Sample size and statistical analysis

Nine previous consecutive accelerometer files from nine ICU patients who underwent CPR showed a mean compression rate (standard deviation, SD) and mean compression depth (SD) of 113.3 (11.4) /min and 6.8 (1.6) cm, respectively. Based on the data, a 20% improvement in adequate chest compression depth, an alpha error of 5% and a power of 0.9, the sample size was calculated to be 29 patients per group.

All data were analyzed using SPSS version 27 (SPSS Inc., Chicago, Illinois, USA). A *P* value < 0.05 was considered as statistically significant. Continuous variables are expressed as mean (SD), and categorical variables were expressed as number (percentage). After testing normal distribution with the Kolmogorov–Smirnov test, normally distributed continuous variables and variables with non-normal distribution and sample size less than 30 were analyzed by Student’s *t*-test and the Mann–Whitney U test, respectively. Analysis of variance test or the Kruskal–Wallis’s test was used to compare continuous variables between four segments, followed by the t-test or Mann–Whitney U test to compare the data between two periods. Chi-square test or Fisher exact tests was used to compare categorical variables between segments. For adjusting multiple comparisons, Bonferroni correction was used.

## Results

**Fig. 1:**
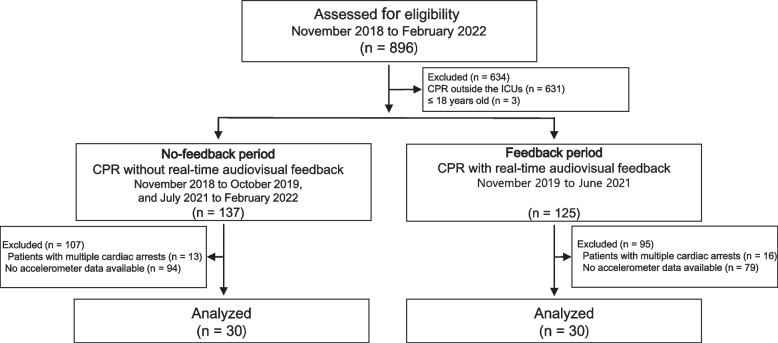
Patient flow chart. N, numbers of cardiopulmonary resuscitation. CPR, cardiopulmonary resuscitation; ICU, intensive care unit

From November 2018 to February 2022, 27,295 compressions in 30 patients in the no-feedback period and 27,965 compressions in 30 patients in the feedback period were collected in both periods (Fig. 1).

There was no significant difference in patient characteristics between the two periods including age, initial rhythm and CPR duration (Table 1). In both periods, 90% of the cardiopulmonary arrest (CPA) occurred in the medical ICU. The most common initial rhythm was pulseless electrical activity, which was 40% in the no-feedback period and 60% in the feedback period. Mean duration of CPR was 21 min in the no-feedback period and 35 min in the feedback period (*P* = 0.07, Table 2). Mean CCRV was significantly faster in the feedback period than that in the no-feedback period (*P* = 0.03, Table 2).

**Table 1:** Demographics and descriptive clinical data of cardiac arrests

	No feedback period (*N* = 30)	Feedback period (*N* = 30)	*P*-value
Demographics			
Age (year)	65.3 (14.6)	67.5 (12.9)	0.54
Sex			0.17
Male	17 (56.7%)	23 (76.7%)	
Female	13 (43.3%)	7 (23.3%)	
Height (cm)	164.1 (9.2)	164.0 (8.3)	0.96
Body weight (kg)	60.2 (12.9)	63.9 (13.7)	0.29
Descriptive clinical data			
APACHE II score	40 (10)	38 (11)	0.50
Cardiac arrest location			1.00
Medical ICU	27 (90.0%)	27 (90.0%)	
Surgical ICU	3 (10.0%)	3 (10.0%)	
Initial rhythm			0.22
Asystole	13 (43.3%)	7 (23.3%)	
Pulseless electrical activity	12 (40.0%)	18 (60.0%)	
Ventricular fibrillation/Pulseless ventricular tachycardia	5 (16.7%)	5 (16.7%)	

Data are presented as mean (standard deviation) or number (%)

*APACHE* acute physiology and chronic health evaluation, *ICU* intensive care unit

**Table 2:** Descriptive data of whole cardiopulmonary resuscitation

	No feedback period (*N* = 30)	Feedback period (*N* = 30)	*P*-value
CPR duration (min)	21.1 (15.1)	35.4 (39.7)	0.07
Chest compression fraction (%)	82.1 (13.9)	81.9 (20.2)	0.95
Mean chest compression depth (cm)	5.7 (1.5)	6.3 (1.3)	0.11
Mean chest compression rate (/min)	110.3 (6.1)	112.0 (6.0)	0.40
Mean chest compression release velocity (mm/sec)	355 (98)	406 (82)	0.03
Defibrillation	7 (23.3%)	8 (26.7%)	0.70
Pre-shock pause (sec)	3.1 (4.1)	1.5 (1.8)	0.69
Post-shock pause (sec)	3.8 (4.6)	1.2 (2.0)	0.23

Data are presented as mean (standard deviation) or number (%)

*CPR* cardiopulmonary resuscitation

The percentage of compressions with both adequate depth and rate in the feedback period was significantly lower in the feedback period compared to that in the no-feedback period (11.8% vs. 16.8%, risk ratio: 0.66, 95% confidential interval: 0.63–0.70, *P* < 0.01) (Table 3). The mean compression depth in the feedback period was 6.7 cm whereas that in the no-feedback period was 6.1 cm (*P* < 0.01). The percentage of chest compressions with adequate depth was 16.9% in the feedback period whereas it was 22.1% in the no-feedback period. In addition, the percentage of compressions with deep depth was significantly higher in the feedback period than the no-feedback period (64.2% vs. 51.4%, *P* < 0.01) (Table 3). The percentage of compressions with adequate rate in the feedback period was significantly lower than that in the no-feedback period (67.3% vs. 75.5%, *P* < 0.01) (Table 3).

**Table 3:** Outcomes of chest compressions for the first 10 min of cardiopulmonary resuscitation

Total compressions	No feedback period (*N* = 27,295)	Feedback period (*N* = 27,965)	*P*-value
Adequate depth and rate	4,584 (16.8)	3,300 (11.8)	< 0.01
Mean compression depth (cm)	6.1 (1.8)	6.7 (1.9)	< 0.01
Compression depth			< 0.01
< 5.0 cm	7,238 (26.5%)	5,285 (18.9%)	
5.0–6.0 cm	6,035 (22.1%)	4,732 (16.9%)	
> 6.0 cm	14,022 (51.4%)	17,948 (64.2%)	
Mean compression rate (/min)	111.9 (11.2)	112.0 (13.0)	0.25
Compression rate			< 0.01
< 100 /min	2,338 (8.6%)	3,330 (11.9%)	
100–120 /min	20,621 (75.5%)	18,816 (67.3%)	
> 120 /min	4,336 (15.9%)	5,819 (20.8%)	
Mean chest compression release velocity (mm/sec)	371 (125)	426 (130)	< 0.01
Chest compression release velocity			< 0.01
≥ 400 mm/sec	10,546 (38.6%)	15,853 (56.7%)	
300–399 mm/sec	8,716 (31.9%)	8,246 (29.5%)	
< 300 mm/sec	8,033 (29.4%)	3,866 (13.8%)	
Return of spontaneous circulation	16 (53.3%)	14 (46.7%)	0.61
ECMO insertion after arrest	2 (6.7%)	6 (20.0%)	0.13
Survival to discharge	1 (3.3%)	0 (0.0%)	0.31

Data are presented as mean (standard deviation) or number (%)

*ECMO* extracorporeal membrane oxygenation

For the CCRV, mean CCRV was significantly faster in the feedback period than that in the no-feedback period (426 mm/s vs. 371 mm/s, *P* < 0.01). In the feedback period, 56.7% of patients were included in a CCRV of 400 mm/s or greater, whereas in the no feedback period, 38.6% of patients were included in that speed. (*P* < 0.01) (Table 3).

**Fig. 2:**
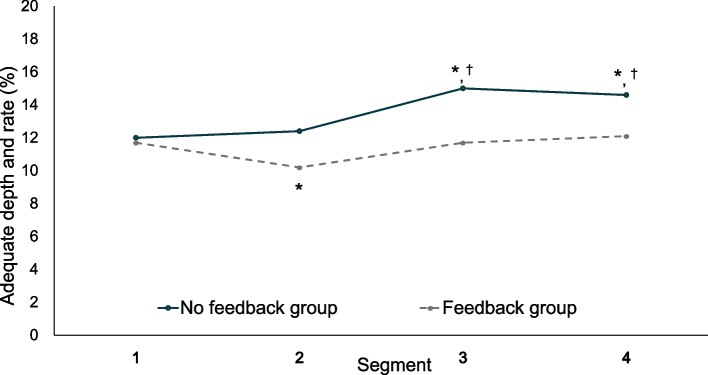
Percentage of adequate depth and rate per segment between the two groups. * P < 0.001 compared with segment 1, ^†^*P* < 0.001 compared with segment 2

We also analyzed the differences in the percentage of compressions with different depth groups, rate groups, and mean CCRV between the segments within a single CPR cycle. There were significant differences between the segments in the percentage of compressions with adequate depth and rate within a single CPR cycle (Fig. 2). In addition, the percentage of compressions with adequate depth and the percentage of compressions with adequate rate were significantly different between the segments within a single CPR cycle in both periods (Supplemental Fig. 1 and 2, Additional file 1 and 2). In both periods, there were significant differences between the segments in mean CCRV within a single CPR cycle (Supplemental Fig. 3, Additional file 3).

## Discussion

We conducted prospective before and after study to evaluate the effect of real-time audiovisual feedback on the quality of chest compression during CPR in the ICUs. Real-time audiovisual feedback did not improve the quality of chest compression.

Compression depth and rate are interactional variables as a faster chest compression rate is related to an impaired chest compression depth [17, 18]. Few studies were conducted to investigate adequate depth and rate of chest compressions simultaneously. In this study, the percentage of compressions with both adequate depth and rate was 16.8% in the no-feedback period. Despite real-time audiovisual feedback, the percentage was not increased in the feedback period. Similar to our results, a previous study of out of hospital cardiac arrest (OHCA) also reported that extended audiovisual feedback did not improve the CPR quality [19]. Numerous studies using feedback systems during simulated CPR reported improvements in CPR quality [20–23]. However, few IHCA studies have shown that the audiovisual feedback system improved the CPR quality, including data such as chest compression depth, and rate [9–11, 24]. Even in studies that showed improvement in CPR quality, the compression rate was improved whereas the depth was not improved [11] or the range of depth was not determined [24]. Therefore, it is difficult to say that the result of improving CPR quality consistently improved both chest compression depth and rate. One of those studies demonstrated that use of a non-AED CPR feedback device (CFA™) significantly improved the adherence to the CPR guidelines based upon chest compression rate, depth, and etc. [10]. The non-AED CPR feedback device is a portable palm-sized device that uses direct pressure measurement contrast to the AED CPR feedback device which uses a calculated pressure based upon movement of an accelerometer. And it provides audio feedback to guide both compression and decompression by clicking sound, so it frees the rescuers to look at the bar graph on the dashboard to figure out the adequate depth and release. The improvement in the adherence to the guidelines including compression depth and rate in the previous study [10] can be explained by the difference in the method of measuring compression pressure and the way of providing feedback.

Low percentage of compressions with both adequate depth and the rate in this study was mainly due to deep compressions of 64% of total compressions in the feedback period. For chest compression depth at a target of 5.0–6.0cm, only 22% was in the target depth range in the no-feedback period in this study, which is similar to previous results of out of hospital cardiac arrests [25, 26]. Chest compressions of  > 5.1cm were known to be associated with improved survival and favorable neurologic outcomes in OHCA [18]. However, chest compression depth greater than 6cm is associated with iatrogenic injury [27]. Thus, it was recommended to avoid excessive chest compression depths for average adults [28]. There are some considerations for deeper chest compressions in the feedback period of this study. First, it is known that the actual depth is less than the depth measured by the accelerometer when compression is performed on the inflatable air mattress and foam mattress used in the ICU bed [29]. Therefore, actual compression depth in our data might be shallower than the calculated depth by accelerometer. Second, chest wall compliance varies among patients [14] and critically ill patients often lose skeletal muscle mass rapidly during the early phase of critical illness [30]. Therefore less muscle mass may lead to increased chest wall compliance resulting deeper compression depth. Third, most of patients have arterial lines for monitoring continuous blood pressure and frequent sampling in the ICUs. And 2020 CPR guidelines suggested that it may be reasonable to use arterial blood pressure if CPR quality can be monitored and optimized [7]. Therefore, the arterial blood pressure might be a guide for compression for the rescuers and CPR leaders in addition to the audiovisual feedback, so it might affect the depth of compression during CPR in our ICUs. Fourth, feedback was provided in English which is not our first language. So, in the critical situation such as CPA, the feedback sound seems like an alarm to rescuers, and it may cause deeper and faster chest compressions in the feedback period. Lastly, there was no audio feedback for the deeper compressions than the recommended depth because audio feedback was only ‘push harder’, and ‘good compression.’ In order to optimize the chest compression, the rescuers should look at the bar graph and depth on the dashboard of the monitor/defibrillator during chest compressions. In 2020 International Consensus on Cardiopulmonary Resuscitation and ECC Science with treatment recommendations, it is proposed to add the role of CPR coach to reduce the workload of the CPR leader and increase chest compression quality.^28^ The CPR coach looks at the dashboard of the monitor/defibrillator and gives continuous verbal feedback to the rescuer, which would allow the rescuer to perform optimal chest compression without constantly looking at the dashboard. Recent pediatric CPR study reported that integration of a CPR coach resulted in an improvement in the percentage of compressions with both adequate depth and rate by 31.8% [31].

In our study, the mean CCF for both periods was 82%, aligning with the recommendation of over 80% from the American Heart Association [32]. Moreover, the mean compression rate for both periods was 110 compressions per minute, which is consistent with the 2015 CPR guidelines recommending a rate of 100–120 compressions/min [13]. However, when categorizing by compression rate, the feedback group had a higher percentage of rates exceeding 120/min compared to the no-feedback period (15.9% in no-feedback vs. 20.8% in feedback). While feedback for a compression rate below 100 is indicated by the sound of a metronome, there is no audible feedback for rates exceeding 120, similar to there being no feedback for depths greater than 6cm. Consequently, compressors may initially speed up in response to the metronome when compressing too slowly, but without a corresponding alert for compressing too quickly, they might consistently maintain a faster rate.

Complete chest wall recoil is an important component of refilling the heart during CPR. Failure to allow complete chest wall recoil is associated with increased intrathoracic pressure and reduced coronary perfusion [33, 34]. And, a previous study showed that fast CCRV (≥ 400 mm/sec) was associated with favorable neurologic outcome and improve survival [35]. In this study, the mean CCRV was significantly faster in the feedback period, and the proportion of CCRV group of  ≥ 400 (mm/s) was significantly higher in the feedback period compared in the no-feedback period. These results can be explained by a higher percentage of compressions with deep depth and a lower percentage of fast rate in the feedback period than those in the no-feedback period.

Finally, to analyze the trend of percentage of compressions with adequate compression depth and rate in a single CPR cycle, we divided each CPR cycle into four segments. In both periods, the percentage of compressions with both adequate compression depth and rate increased significantly in the fourth segment than in the second segment, mainly due to the decrease of the percentage of deep compressions in the fourth segment. And in the no-feedback period, the ratio of deep chest compression exceeding 6 cm significantly decreased over the segments. On the other hand, no prominent decreasing trend was seen in the feedback period. This can be considered that the feedback gave the effect of maintaining the compression depth and rate over time in a single CPR cycle.

There are a number of limitations in this study. First, the quality of chest compressions before applying CPR pads and an accelerometer sensor could not be collected. However, the interval would have been very short since the monitor/defibrillator and the sensor were placed along with CPR immediately when the patient was in CPA. Second, we did not know the exact compression depth although we used backboards during the CPR. Additionally, we could not consider all the variability of compliance of patients’ chest walls and lungs. But we used the same type of bed mattresses, backboard and accelerometer in ICUs, so we minimized the mechanical variability due to the devices. And we did not record assisted ventilation information such as frequency and tidal volume. Third, more than seventy percent of CPRs in ICUS were not included in this data mainly due to unavailable accelerometer files. This could lead selection bias. However, monthly distribution of CPRs and recorded data were similar. Fourth, we did not enroll patients with predominantly cardiac disease who were admitted to a cardiac ICU. So, it is difficult to generalize our results to cardiac ICU patients. Lastly, this study was not powered to detect differences in ROSC or survival. Larger trials are needed to evaluate those clinical outcomes.

## Conclusion

Real-time audiovisual feedback system did not enhance CPR quality based on our results. It exhibited a higher percentage of compression depth deeper than the recommended depth of 5.0–6.0 cm and an increased rate of compressions faster than the advised 100–120/min. It is inevitable that a certain degree of variability in the results may occur in actual clinical settings. Therefore, our results should not be interpreted as discouraging the use of audiovisual feedback in real CPR situations. On the contrary, it's important to investigate better methods for integrating feedback in actual clinical scenarios to enhance CPR effectiveness.

### Supplementary Information

**Additional file 1: Supplemental figure 1.** Percentage of depth groups per segment within a single cycle. A: no-feedback period, B: feedback period A. * *P* < 0.001 compared with segment 3 and 4, †*P* = 0.002 compared with segment 3, ‡*P* < 0.001 compared with segment 4, §*P* = 0.007 compared with segment 4. B. * *P* < 0.001 compared with segment 2, †*P* = 0.002 compared with segment 3, ‡*P* = 0.004 compared with segment 4.

**Additional file 2:** **Supplemental figure 2.** Percentage of rate groups per segment within a single cycle. . A: no-feedback period, B: feedback period A. * *P* < 0.001 compared with segment 1, 2, and 3, †*P* < 0.001 compared with segment 3 and 4. B. * *P* < 0.001 compared with segment 2, 3, and 4, †*P* < 0.001 compared with segment 3 and 4.

**Additional file 3:** **Supplemental figure 3.** Mean chest compression release velocity between the two groups within a single cycle. White bar represents CCRV in the no-feedback period, black bar represents CCRV in the feedback period, * *P* < 0.05. CCRV, chest compression release velocity.

## Data Availability

The datasets used and/or analyzed during the current study are available from the corresponding author on reasonable request.

## Abbreviations

CPR: Cardiopulmonary resuscitation
ECC: Emergency Cardiovascular Care
ROSC: Return of spontaneous circulation
AED: Automated external defibrillator
IHCA: In-hospital cardiac arrests
VF: Ventricular fibrillation
VT: Pulseless ventricular tachycardia
PEA: Pulseless electrical activity
ICU: Intensive care unit
CCF: Chest compression fraction
CCRV: Chest compression release velocity
OHCA: Out of hospital cardiac arrest
