# Supplementary material for: The effect of audiovisual feedback of monitor/defibrillators on percentage of appropriate compression depth and rate during cardiopulmonary resuscitation
Source: BMC Anesthesiol. 2023 Oct 5;23:334. doi: 10.1186/s12871-023-02304-9 (PMC10552289; doi:10.1186/s12871-023-02304-9)
Supplement: Supplementary file 1 — Additional file 1: Supplemental figure 1. Percentage of depth groups per segment within a single cycle. A: no-feedback period, B: feedback period A. * P < 0.001 compared with segment 3 and 4, †P = 0.002 compared with segment 3, ‡P < 0.001 compared with segment 4, §P = 0.007 compared with segment 4. B. * P < 0.001 compared with segment 2, †P = 0.002 compared with segment 3, ‡P = 0.004 compared with segment 4. [file 12871_2023_2304_MOESM1_ESM.pdf]

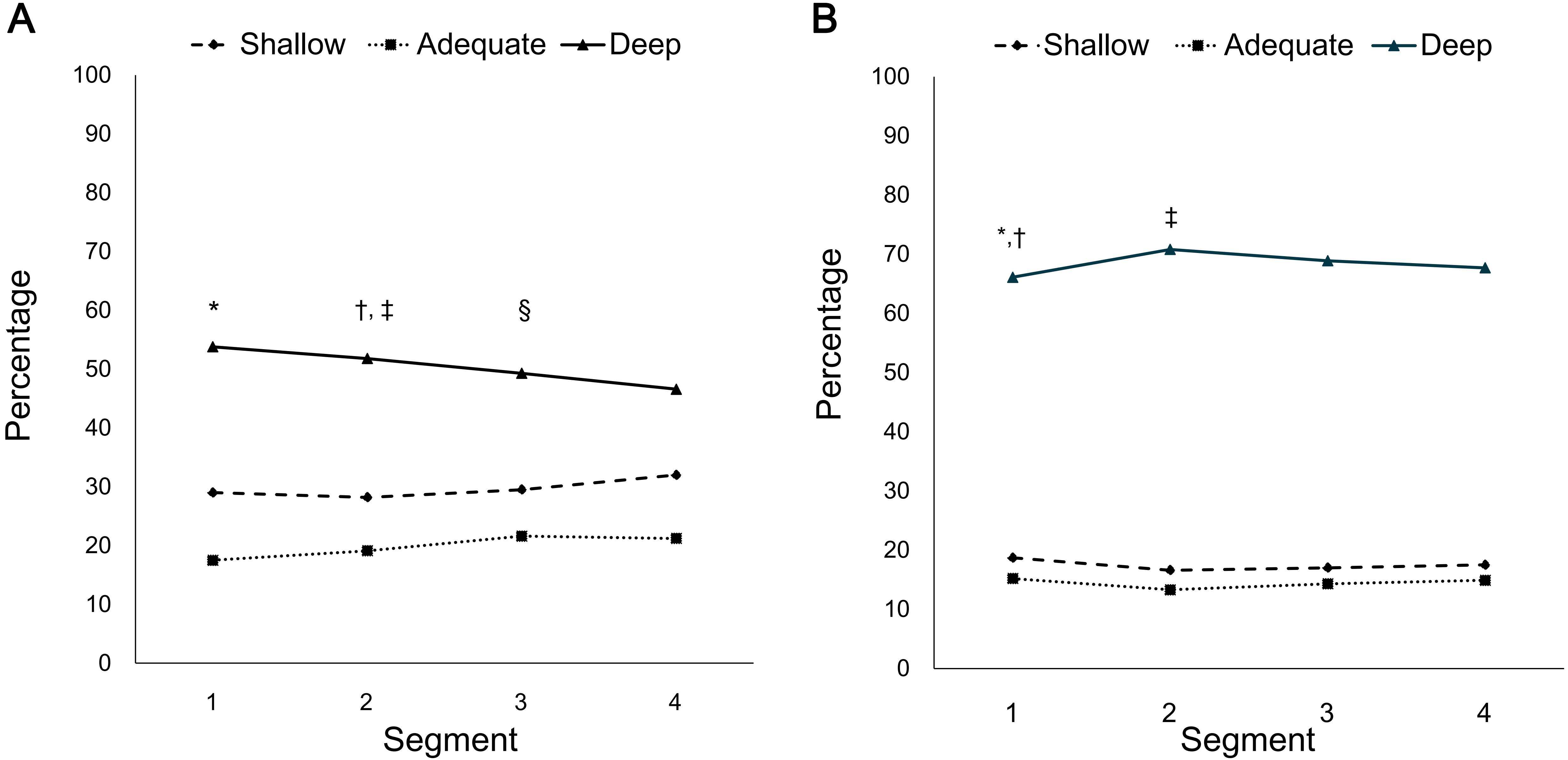

Supplemental figure 1. Percentage of depth groups per segment within a single cycle. A: no-feedback period, B: feedback period

A. * $P < 0.001$ compared with segment 3 and 4, †$P = 0.002$ compared with segment 3, ‡$P < 0.001$ compared with segment 4, §$P = 0.007$ compared with segment 4. B. * $P < 0.001$ compared with segment 2, †$P = 0.002$ compared with segment 3, ‡$P = 0.004$ compared with segment 4.
