# Supplementary material for: The effect of audiovisual feedback of monitor/defibrillators on percentage of appropriate compression depth and rate during cardiopulmonary resuscitation
Source: BMC Anesthesiol. 2023 Oct 5;23:334. doi: 10.1186/s12871-023-02304-9 (PMC10552289; doi:10.1186/s12871-023-02304-9)
Supplement: Supplementary file 2 — Additional file 2: Supplemental figure 2. Percentage of rate groups per segment within a single cycle. . A: no-feedback period, B: feedback period A. * P < 0.001 compared with segment 1, 2, and 3, †P < 0.001 compared with segment 3 and 4. B. * P < 0.001 compared with segment 2, 3, and 4, †P < 0.001 compared with segment 3 and 4. [file 12871_2023_2304_MOESM2_ESM.pdf]

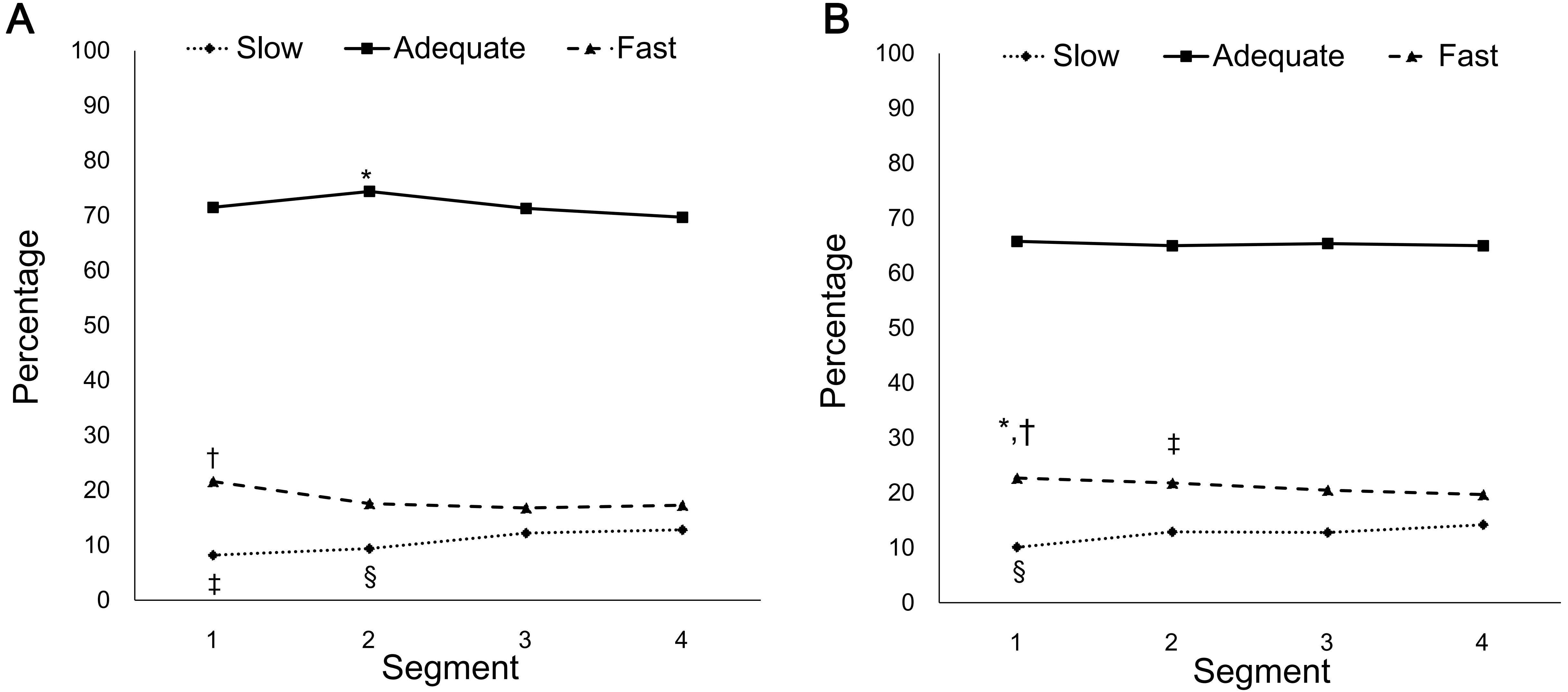

Supplemental figure 2. Percentage of rate groups per segment within a single cycle. . A: no-feedback period, B: feedback period

A. * *P* < 0.001 compared with segment 1, 2, and 3, †*P* < 0.001 compared with segment 3 and 4. B. * *P* < 0.001 compared with segment 2, 3, and 4, †*P* < 0.001 compared with segment 3 and 4.
