# Supplementary material for: The effect of audiovisual feedback of monitor/defibrillators on percentage of appropriate compression depth and rate during cardiopulmonary resuscitation
Source: BMC Anesthesiol. 2023 Oct 5;23:334. doi: 10.1186/s12871-023-02304-9 (PMC10552289; doi:10.1186/s12871-023-02304-9)
Supplement: Supplementary file 3 — Additional file 3: Supplemental figure 3. Mean chest compression release velocity between the two groups within a single cycle. White bar represents CCRV in the no-feedback period, black bar represents CCRV in the feedback period, * P < 0.05. CCRV, chest compression release velocity. [file 12871_2023_2304_MOESM3_ESM.pdf]

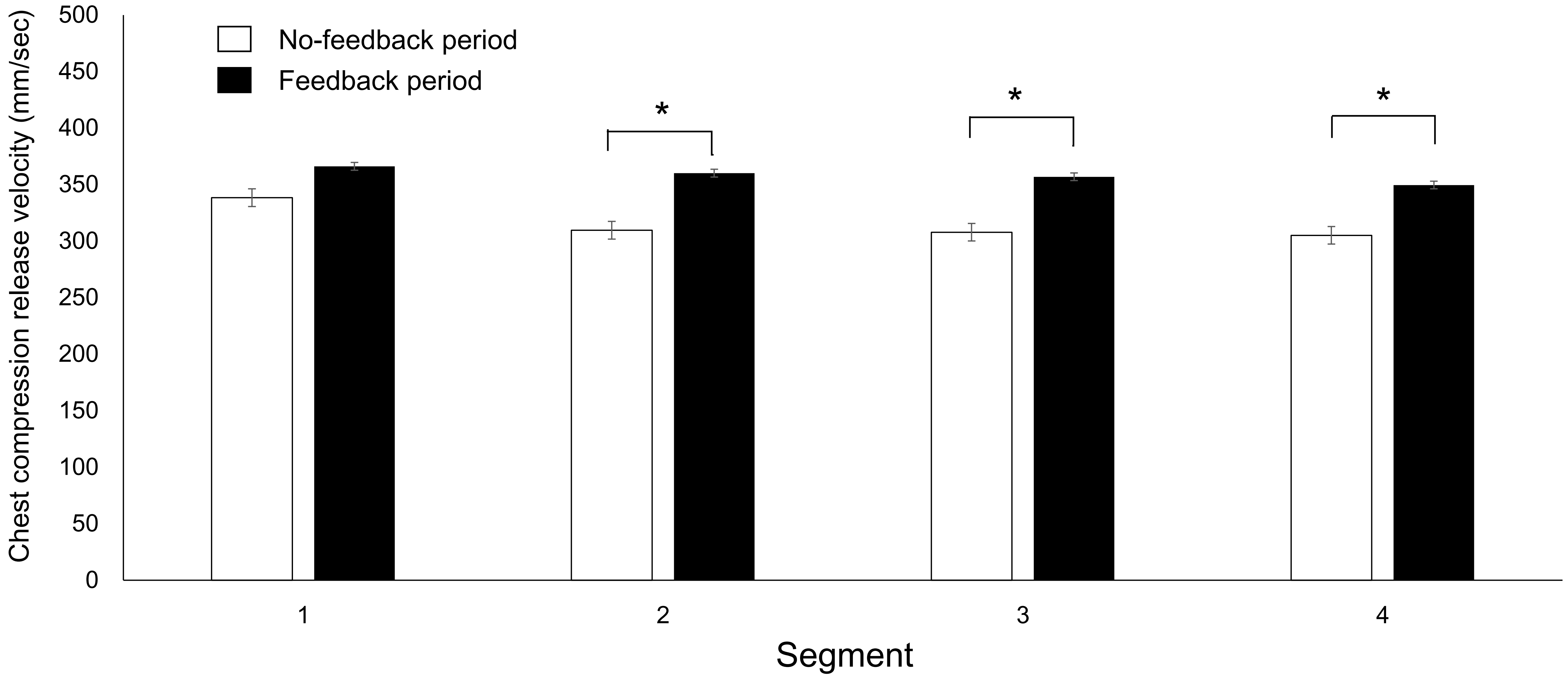

Supplemental figure 3. Mean chest compression release velocity between the two groups within a single cycle.

White bar represents CCRV in the no-feedback period, black bar represents CCRV in the feedback period, * $P < 0.05$. CCRV, chest compression release velocity.
